# Allocating colorectal cancer patients to different risk categories by using a five-biomarker mRNA combination in lymph node analysis

**DOI:** 10.1371/journal.pone.0229007

**Published:** 2020-02-12

**Authors:** Lina Olsson, Marie-Louise Hammarström, Anne Israelsson, Gudrun Lindmark, Sten Hammarström

**Affiliations:** 1 Department of Clinical Microbiology, Section of Infection and Immunology, Umeå University, Umeå, Sweden; 2 Department of Clinical Sciences, Lund University, Helsingborg, Sweden; Bilkent University, TURKEY

## Abstract

**Background and aims:**

Curative surgery saves ≈50% of all patients with colorectal cancer (CRC) while remaining patients have synchronous or will develop metachronous metastases. Presently, the single most important prognostic factor is histopathological detection of disseminated tumor cells in regional lymph nodes. However, the routine method has several limitations. The aim was to identify biomarker mRNAs that could be combined in a formula that would allow better prediction of patients' survival after surgery.

**Methods:**

Screening for biomarker mRNAs overexpressed in CRC was performed by genome-wide hybridization bead array, with verification by qRT-PCR. Specific qRT-PCR assays with copy standards were developed for 5 selected genes and mRNA expression levels determined in lymph nodes from 174 CRC patients (517 nodes) and 24 control patients (118 nodes). Prognostic value of biomarker mRNAs was estimated. A cut-off was set using univariate Cox regression analysis and used for calculation of differences between patient groups in disease-free survival 12 years after surgery (Kaplan-Meier survival model) and risk for recurrent disease (Cox's regression analysis). A formula was constructed for evaluation of the prognostic value of the biomarkers in combination.

**Results:**

Two new biomarkers, SLC35D3 and POSTN with prognostic value were identified. SLC35D3 was expressed in the epithelium derived tumor cells and POSTN in fibroblasts. Combined with CEACAM5, KLK6 and MUC2 they could be used to identify risk groups. A formula was constructed using CEACAM5 as denominator for KLK6, SLC35D3 and MUC2 and 18S rRNA as denominator for POSTN. The formula yielded 5 categories (-1, 0, 1, 2, 3). Categories (-1 and 0) had good prognosis, categories (1 and 2) relatively poor prognosis and category (3) very poor prognosis.

**Conclusion:**

Lymph node analysis using 5 selected biomarker mRNAs and 18S rRNA in combination allowed allocation of CRC patients to different risk categories with respect to recurrent disease.

## Introduction

Colorectal cancer (CRC) is the third most diagnosed form of cancer globally and the second leading cause of mortality among cancer patients. CRC causes a huge burden on the healthcare systems. The single most important prognostic factor in patients with CRC is presence or absence of tumor cell metastasis in regional lymph nodes [1–3]. Approximately 50% of patients with tumor-cell-positive lymph nodes, i.e. stage III CRC (anyTN1-2M0) and about 25% with no detected tumor-cell-positive lymph nodes, i.e. stage I (T1-2 N0M0) and stage II (T3-4N0M0) patients will recur after curative surgery [4–6]. Presence or absence of lymph node metastasis is generally determined by histopathological examination of hematoxylin and eosin (H&E) stained tissue sections of resected regional lymph nodes. Present guidelines require that at least 12 lymph nodes should be examined [6–8]. In the TNM classification, N1 signifies that 1 to 3 nodes were positive for presence of tumor cells and N2 that 4 to 6 nodes were positive. N2 patients have poorer prognosis than N1 patients [9]. Refinement of the N staging system for CRC is still on-going [10].

The observation that about 25% of stage I and II patients develop distant metastases, and the established relationship between lymph node metastases and prognosis, indicate that tumor cells can escape detection by histopathological examination of regional lymph nodes. The fact that only a small volume of the lymph node can be examined in clinical practice (at best a few percent) and inadequate sensitivity of the H&E method are probably the main reasons for misclassification of CRC patients. This may lead to that these will not be offered adjuvant chemotherapy, which most likely would have been beneficial. For a more accurate assessment of presence or absence of tumor cells in lymph nodes, molecular methods may be used for analysis, extracting the entire lymph node or, presently, half the node thereby complementing and allowing comparison to histopathology. Real-time quantitative reverse transcriptase-polymerase chain reaction (qRT-PCR) analysis is a most useful method for assessment of biomarker mRNA expression as proxy for disseminated tumor cells. It has been used successfully for biomarkers such as carcinoembryonic antigen cell adhesion molecule 5 (CEACAM5), kallikrein related peptidase 6 (KLK6), cytokeratin 20 (CK20) and guanylate cyclase 2C (GUCY2C) [6,11–14].

In this study we investigate the utility of a combination of selected biomarker mRNAs for lymph node analysis in order to more precisely define the risk of developing recurrent disease and propose a formula for calculating relative risk. Biomarkers were selected to reveal different properties of the CRC tumor cells and of the tumor cell environment.

## Materials and methods

### Patients

Surgery for CRC was carried out in 174 patients [87 men, 87 women, median age 72, (range 51–90) years]. Sixteen of the tumors were located in rectum and 158 in colon. Seven of the rectal cancer patients received 25 Gy of preoperative radiotherapy. A locally radical tumor resection was carried out in all patients. Tumors were divided into the two categories, poorly differentiated high-grade and moderately/well differentiated low-grade according to the recommendations of the Royal College of Pathologists' Dataset for histopathological reporting of colorectal cancer [15]. Twenty-three tumors were of high-grade and 137 of low-grade. Information about grade was missing for 14 tumors. According to the TNM classification, 30 patients were in stage I (T1-2N0M0), 79 in stage II (T3-4N0M0), 47 in stage III (anyTN1-2M0) and 18 in stage IV (anyTanyNM1). N-classification was based on routine histopathology examination of H&E stained sections of 2,421 lymph nodes carried out by pathologists in the clinics at Norrland University Hospital, Umeå, Sweden and Helsingborg Hospital, Helsingborg, Sweden. A median of 13 (range 1–51) lymph nodes per patient was examined. Thirty-four patients (4 in stage II, 19 in stage III and 11 in stage IV) received chemotherapy after surgery. The median follow-up time was 75 (range 33–147) months. No patient was lost at follow-up. Controls included 18 men and 5 women [median age 25 years, (range 10–61)] undergoing surgery for ulcerative colitis (UC; n = 18), Crohn’s colitis (n = 3), colon lipoma (n = 1) and rectal prolapse (n = 1).

### Lymph nodes

Lymph nodes were retrieved from the resected specimens and bisected with separate, sterile knives. One half of each node was included in routine histopathology examination, i.e. fixed in 10% buffered formalin and embedded in paraffin for sectioning and H&E-staining. The other half was snap frozen in liquid nitrogen and stored at -70°C until RNA extraction [16]. From CRC patients, 517 lymph nodes (91, 261, 115 and 50 nodes from stage I-IV patients, respectively) were collected. A median of 2 (range 1–15) lymph nodes was obtained per patient. From control patients, 118 lymph nodes (85, 16, 13 and 4 nodes from UC, Crohn’s colitis, colon lipoma and rectal prolapse patients, respectively) were collected.

### Primary and distant CRC tumors and normal colon tissue

Primary tumors from 84 CRC patients were analyzed (16 stage I patients, 35 stage II patients, 25 stage III patients and 8 stage IV patients). Primary tumor stage distribution (pT1-pT4) was 2, 14, 55 and 13. The tumor differentiation grade was poor, moderate and high in 11, 70 and 3 tumors, respectively. The tumor samples, approximately 0.5 x 0.5 x 0.5 cm in size, were collected immediately after resection, snap-frozen, and stored in -70°C until RNA extraction. Five normal colon samples retrieved from the proximal or distal resection margin and two distant liver metastases samples were also collected and treated accordingly.

### Epithelial cells from colon tissue

Colonic intestinal epithelial cells (iECs) were isolated from the normal colon mucosa retrieved from the resection margins of colon cancer (CC) patients at operation as described [17,18].

### Cell lines and peripheral blood mononuclear cells

Total RNA from the human cell lines LS174T, HT29, T84, HCT8 and Caco2 (all colon carcinomas from American Type Culture Collection (ATCC), Rockville, MD, USA), Jurkat and Molt-4 (T cell lymphomas from Deutsche Sammlung von Mikroorganismen und Zellkulturen (DSMZ), Braunschweig, Germany), CNB6 and KR4 (EBV-transformed B cell lines; kind gifts from Dr P. Sideras, BRFAA, Athens, Greece), U266 (plasmacytoma cell line; from DSMZ), U937 (monocyte-like cell line; from ATCC), K562 (erythroblastoid cell line; from ATCC), HL60 (granulocyte cell line; from ATCC) and FSU (primary foreskin fibroblasts; kind gift from Prof. G. Wadell, Umeå University, Sweden) was analyzed [12]. Peripheral blood mononuclear cells (PBMCs) were isolated from healthy adults by Ficoll-Isopaque gradient centrifugation, and RNA extracted directly and after polyclonal activation as described [12].

### RNA preparation

Total RNA was extracted using the acid guanidine phenol chloroform method as described [12], dissolved in RNase-free water containing rRNasin ribonuclease inhibitor (Promega, Madison, WI, USA) and stored at -70°C until gene expression analysis by genome-wide hybridization bead array screening and qRT-PCR analysis.

### Gene expression analysis using genome-wide hybridization bead array screening of cRNA libraries

Concentrations and purity of total RNA samples were determined by measuring optical density at 260 nm (OD260) and OD280 in a NanoDrop ND-1000 spectrophotometer V3.0.0 (NanoDrop Technologies, Wilmington, DE, USA). Four-hundred ng of total RNA of each sample subjected to analysis was converted to biotinylated double-stranded cRNA according to the Illumina Totalprep RNA Amplification Kit (Ambion, Austin, TX, USA). The procedure yielded > 15 μg cRNA and the purity estimated as OD260/OD280 was ≥1.8. Agarose gel analysis of sample integrity in a 2100 Bioanalyser (Agilent Technologies, Palo Alto, CA, USA) showed cRNAs suitable for hybridization with normal distribution of fragments between 200 and 6,000 base pairs in length. The labeled cRNA samples were then hybridized on Sentrix HumanRef-8_V2 Expression Beadchips (Illumina, San Diego, CA, USA) incubated with streptavidin-Cy3 and scanned using the Illumina Beadstation GX (Illumina).

Results were analyzed by using Illumina Beadstudio software (version 3.3) for direct hybridization assays. Intensity data were normalized by Beadstudios cubic spline algorithm with subtracted background. Significant difference in expression was calculated using the Beadstudio software Error Model Illumina Custom with multiple testing corrections using Benjamini and Hochberg False Discovery Rate (FDR) [19]. Difference in gene expression was calculated as fold change, dividing the signal in individual CRC samples of interest over the average signal of controls. Data-files from hybridization bead array were submitted to Gene Expression Omnibus (GEO) DataSets (www.ncbi.nlm.nih.gov/geo) with accession number GSE141174.

### Gene expression analysis using real-time qRT-PCR

Quantification of mRNAs was done in total RNA using Taqman Gene Expression Assays (Applied Biosystems, Foster City, CA, USA; S1 Table) and inhouse constructed real-time qRT-PCR assays with RNA copy standards. The 3'-primer was used as template for recombinant thermostable *Thermus thermophilus* (Tth) DNA polymerase (Applied Biosystems) or Tth polymerase in the LightCycler 480 RNA master hydrolysis probes kit (Roche, Mannheim, Germany) for gene specific reverse transcription in each qRT-PCR run and the qPCR step run with primers placed in different exons and a reporter dye marked probe placed over the exon boundary. Emission from the released reporter dye was measured by the ABI Prism 7700 Sequence Detection System (Applied Biosystems).

Real-time qRT-PCR assays with RNA copy standards were constructed for CEACAM5, KLK6, mucin 2 (MUC2), solute carrier family 35 member D3 (SLC35D3) and periostin (POSTN) as described [16,18]. Primer and probe sequences were: CEACAM5, forward primer 5´-CTGATATAGCAGCCCTGGTGTAGT-3´, reverse primer 5´-TGTTGCAAATGCTTTAAGGAAGA-3´ and probe 5´-TTCATTTCAGGAAGACTGACAGTTGTTTTGCTT-3´; KLK6, forward primer 5´-CTTATCCATCCACTGTGGGTC-3´, reverse primer 5´-TGGATCACAGCCCGGA-3´and probe 5´-CACTGCAAAAAACCGAATCTTCAGGTC-3´; MUC2, forward primer 5´-CCGGGCTGCTCATTGAGA-3´, reverse primer 5´-TAGTGTCCAGCTCCAGCATGA-3´ and probe 5´-TCCCGGTTCCACATGA-3´; SLC35D3, forward primer 5´-TCATCACCACCTGCGGC-3´, reverse primer 5´-AGCACTCCCGTGACGTACC-3´ and probe 5´-CCTGGCAGGAGCCGGCGA-3´; POSTN, forward primer 5´-ACAGCTCAGAGTCTTCGTATATCG-3´, reverse primer 5´-CCCTTGCTTACTCCCTTTCTC-3´and probe 5´-ACAGCTGTCTGCATTGA-3´. The reporter dye at the 5´-end of each probe was FAM. The quencher dye at the 3´-end was TAMRA for CEACAM5, KLK6 and SLC35D3 and MGB for MUC2 and POSTN. Serial dilutions of the RNA copy standard at concentrations from 10^3^ to 10^8^ copies/μl were included in each qRT-PCR run. Concentrations in unknown samples were determined from the standard curve and expressed as copies of mRNA/μl. All qRT-PCR analyses were carried out in triplicates. The reproducibility of the assays was determined as described [12]. The coefficient of variation (CV) based on PCR ct-values was 1.0–1.8%. The mean CV for estimation of mRNA content covering 10^3^ to 10^8^ RNA copies per reaction was 16% for MUC2, 25% for CEACAM5 and 27% for KLK6.

The concentration of 18S rRNA was determined in each sample by real-time qRT-PCR for normalization of mRNA levels as described [12,20]. All samples contained >25 U 18S rRNA per reaction mixture.

Results from qRT-PCR analyses with RNA copy standard are expressed as mRNA copies per unit of 18S rRNA. Results from qRT-PCR analyses with Taqman Gene Expression Assays are expressed as relative quantity (RQ) where the mRNA concentrations are normalized to the18S rRNA concentration in the same sample by calculating the ΔCT between the CT for the mRNA species and the CT for 18S rRNA and RQ calculated as 2^(-ΔΔCT)^ where ΔΔCT is Δ CT for the sample minus the ΔCT values of one lymph node of one Crohn's colitis patient.

### Statistical analysis

Correlation between biomarker mRNA levels was analysed using the non-parametric Spearman correlation coefficient. Differences in biomarker mRNA levels between two patient groups were analysed by two-tailed Mann-Whitney U test. Descriptive values of mRNA levels are given as median and range or interquartile range from the 25^th^ to the 75^th^ percentile (IQR). The software utilized for these statistical calculations was GraphPad Prism 6 (Graphpad Software, San Diego, CA, USA).

The SPSS (IBM Corporation, Armonk, NY, USA) was used for statistical analyses of differences in disease-free survival and risk for recurrent disease after surgery between patient groups were calculated according to Kaplan-Meier survival model in combination with the log rank test and univariate and multivariate Cox's regression analysis. Patients who died from causes other than CRC were considered as disease free. Descriptive values of risk and survival time are given as mean and 95% confidence interval (CI).

A *P*-value < 0.05 was considered statistically significant.

### Ethics statement

All procedures involving human participants were in accordance with the ethical standards of the institutional research committee and with the 1964 Helsinki Declaration and its later amendments or comparable ethical standards. Tumor samples and lymph nodes were collected after patients’, and in one case his parents, written, informed consent and blood samples from healthy donors for isolation of PBMCs were taken after oral informed consent. The study was approved by the Local Ethics Research Committee of the Medical Faculty, Umeå University, Umeå, Sweden (Registration number: 03–503; date of approval: 2003-12-03).

## Results

### Selection of biomarker mRNAs for detection of lymph node metastases in patients with colorectal cancer

**Fig 1:**
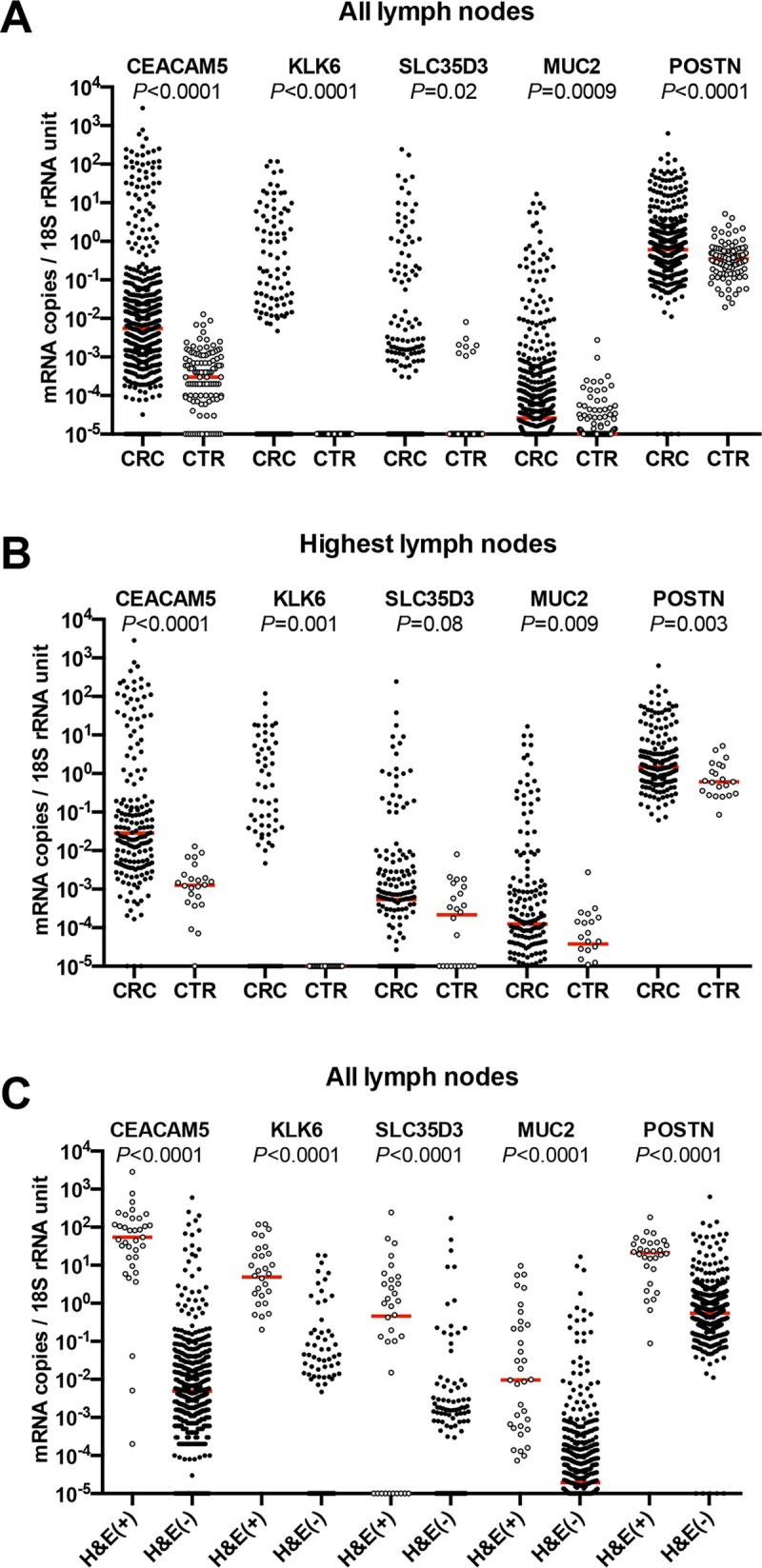
Biomarker mRNA expression levels in lymph nodes from CRC patients (CRC) and Controls (CTR). (**A**), All lymph nodes. Five hundred and seventeen lymph nodes of CRC patients were analyzed for CEACAM5 mRNA, 503 for KLK6 mRNA, 479 for SLC35D3 mRNA, 509 for MUC2 mRNA and 492 for POSTN mRNA. (**B**), Highest lymph nodes. One hundred seventy-four lymph nodes of CRC patients were analyzed for CEACAM5 mRNA, 166 for KLK6 mRNA, 166 for SLC35D3 mRNA, 172 for MUC2 mRNA and 166 for POSTN mRNA. (**C**), All hematoxylin & eosin positive [H&E(+)] compared to all H&E(-) lymph nodes of CRC patients. Thirty five H&E(+) and 482 H&E(-) lymph nodes were analyzed for CEACAM5 mRNA. The number of nodes analyzed for KLK6 mRNA, SLC35D3 mRNA, MUC2 mRNA and POSTN mRNA were 32 plus 471, 34 plus 445, 34 plus 476 and 30 plus 462, respectively. (**A**–**C**), 10^−5^ mRNA copies/18S rRNA unit collects lymph nodes with expression levels of 10^−5^ mRNA copies/18S rRNA unit and below. Each dot represents one lymph node. Red horizontal lines indicate median values. *P*-values from pairwise comparisons of the expression levels in the CRC versus CTR and the H&E(+) versus H&E(-) groups are given above each pair of columns.

The following strategy was used for identification of new biomarker mRNAs of prognostic value when expressed in lymph nodes: cRNA libraries of 4 lymph nodes with disseminated tumor cells detected by routine histopathology [H&E(+)] from stage III and IV CC patients and 3 primary tumors from 3 stage III CC patients (two T3 and one T4) were compared individually against a panel of cRNA libraries of control tissues in genome-wide hybridization bead array analysis. Control tissues were lymph nodes from 2 UC patients, 1 lipoma patient, 1 Crohn's colitis patient and isolated normal iECs from 3 CC patients. To qualify for further analyses the mRNA signal of the particular biomarker should be at least 5-fold higher than the mean signal for the same mRNA species in the control samples with a *P*-value <0.05. Moreover, these criteria should be fulfilled for at least 5 of the cancer tissue samples. Forty-six mRNA species fulfilled these criteria (S2 Table) of which 14 mRNA species did so in all 7 cancer tissue samples. The following criteria were used to select biomarker mRNAs from the list of 46 overexpressed genes (S2 Table): 1) Signals should be higher in 3 new individual primary CC tumors and 3 new normal colon iEC samples in hybridization bead array; 2) Genes should preferentially be overexpressed in 7/7 or 6/7 tumors; 3) Genes should not be expressed at high levels in lymph nodes or immune cell line lines as revealed by the information in the database "Human Protein Atlas" [21] and in many cases experimentally verified by us; 4) Survival data for CRC patients, as reported in the "Human Protein Atlas" in which mRNA levels in the primary tumors had been determined for the gene. Data should indicate some survival difference between high and low expressers; 5) Genes that code for proteins associated with other diseases or conditions than CRC should preferably be excluded. Based on these five criteria we selected 11 genes, that met most of them, for further study. The selected 11 genes plus CEACAM5 and C6orf223 were analyzed using commercially available qRT-PCR assays (S1 Table) and the in-house constructed assays described in the Materials and methods section. For each of the different cell types and tissues we also determined the 18S rRNA concentration to allow normalization of expression levels. The RQ-value of each cell type and tissue was calculated using a lymph node sample from one Crohn's colitis patient for normalization. The RQ values for the 13 selected genes are given in S3 Table. According to definition the lymph node from the Crohn's colitis patient gives a RQ value of 1.0. To categorize the different pattern into groups we performed correlation analysis between the different genes (S4 Table). Three major groups were identified: A) the CEACAM5/KLK6 group with members CEACAM5, KLK6, SERPINB5, FOXQ1, CLDN2, AZGP1, CDH3 and C6orf 223; B) the POSTN group with members POSTN and SULF1; C) the PNCK group with members PNCK and C16orf 59. SLC35D3, finally was not clearly grouped but seemed loosely associated to the A group. The POSTN group stands out. It shows a pattern of expression which indicates that the two members preferentially are expressed in fibroblasts but not in the CC tumor cell lines or normal colon iECs. Still they are expressed at high levels in H&E(+) lymph nodes (Fig 1C) possibly indicating that POSTN is a marker for fibroblasts/fibroblast-like cells supporting the cancer cells. It was selected for further studies. In the CEACAM5/KLK6 group KLK6 stands out as the most specific and interesting as a marker for CC cancer epithelial cells. It has been analyzed in a previous publication and been shown to add clinical information over analysis of CEACAM5 identifying tumor cells that are particularly aggressive [13]. It was also selected for further research. SLC35D3 mRNA was selected because it showed high CC specificity but was different from members of the CEACAM5/KLK6 group. In addition, CEACAM5 was selected because it appears to be expressed in all primary tumors and generally at high levels [12,22,23] and MUC2 because mucinous colorectal adenocarcinoma has a better prognosis than adenocarcinoma in general [12,24].

### Expression levels of CEACAM5, KLK6, SLC35D3, MUC2 and POSTN mRNAs in different cancerous and normal human tissues and cell lines

The mRNA expression levels of CEACAM5, KLK6, SLC35D3, MUC2 and POSTN in 18 different human tissues and cell lines are shown in Table 1. The results are given as mRNA copies/18S rRNA unit using in-house designed qRT-PCR systems with RNA copy standards. In comparison to the expression levels in primary CC tumors none of the six immune cell lines or resting or activated PBMCs expressed appreciable levels of any of the five biomarker mRNAs. Moreover, while KLK6 and SLC35D3 were CC-tumor specific in the sense that only CC tumor tissue expressed the biomarkers compared to normal colon tissue or normal colon epithelial cells, CEACAM5 and MUC2 were expressed at approximately the same levels in normal and cancerous colon tissues. POSTN, finally, was confirmed as a marker for fibroblasts and was expressed in the primary foreskin fibroblasts and in primary tumors, liver metastases and normal colon tissue but not in CC tumor cell lines.

**Table 1:** Expression levels of CEACAM5, KLK6, SLC35D3, MUC2, and POSTN mRNAs in primary CRC tumors, normal colon, normal colon epithelial cells, CC cell lines, peripheral blood mononuclear cells, immune cell lines, a fibroblast cell line, CC liver metastases and normal liver.

			mRNA copies / 18S rRNA unit				
SOURCE		n°	CEACAM5	KLK6	SLC35D3	MUC2	POSTN
Primary CRC		57	176*	2.9	0.1	1.1	9.7
tumors			(112–281)**	(0.9–8.5)	(0.04–0.4)	(0.3–4.2)	(4.6–22.2)
CC cell	LS174T	1	328^#^	79	0^##^	4.3	0
lines	HT29	1	32	256	0.02	0.01	0
	T84	1	33	316	0.7	0.5	0
	HCT8	1	32	32	0.07	0.02	0
	CaCo2	1	3	0.4	0.09	0.04	0.0009
Normal	Tissue	5	222*	0	0.02	9	5.9
colon	iECs	5	300*	0	0.0009	32	0.2
PBMCs	Fresh	1	0	0	0.06	0	0
	Act.	1	0	0	0	0	0
Immune cell lines							
T cell	Jurkat	1	0	0	0	0	0.009
B cell	CNB6 + KR4	1	0	0	0	0	0
Plasma cell	U266	1	0	0	0	0	0.005
Monocyte	U937	1	0.005	0	0	0	0
Granulocyte	HL60	1	0	0	0	0	0
Other cell lines							
Erythroid	K562	1	0	0	0.09	0	0.001
Fibroblasts	FSU	1	0.0002	0	0	0.004	5.5
Liver tissue							
Metastasis		2	78	2	0.07	0.003	22.6
Normal		2	0.01	0	0	0.00004	2.3

° n = number of samples.

* Median of indicated number of samples.

** Interquartile range from the 25^th^ to the 75^th^ percentile.

^#^ Cell lines and PBMCs: mean of 3 independent determinations.

^##^ 0, < 0.00001 mRNA copies/18S rRNA unit.

iECs, isolated epithelial cells; PBMCs, peripheral blood mononuclear cells. The T-stages of the 57 primary tumors were: T2, 11; T3, 39; T4, 7.

### Biomarker mRNA expression levels in lymph nodes from CRC patients and controls

Regional lymph nodes obtained at surgery from 174 CRC patients and 24 control patients were analyzed. Each patient contributed several lymph nodes and in total 517 and 118 lymph nodes from CRC patients and controls, respectively were studied. The result is shown in Fig 1. In Fig 1A and 1C the values for all lymph nodes are given and in Fig 1B the value for the lymph node giving the highest value for each patient is given. In Fig 1C the biomarker mRNA expression levels in H&E(+) and H&E(-) lymph nodes are compared. As can be seen a large fraction of CRC lymph nodes express biomarker mRNAs at higher levels than the controls. The fraction is largest for CEACAM5 and KLK6 and smallest for POSTN. Moreover, for CEACAM5, KLK6, SLC35D3 and POSTN almost all H&E(+) lymph nodes show high biomarker mRNA levels. Interestingly, a substantial fraction of H&E(-) nodes also showed high biomarker levels. The distribution of values for MUC2 in H&E(+) lymph nodes was broad from very high to low values but still were above the levels of controls (Fig 1A and 1C).

The fraction of stage I to IV CRC patients expressing biomarker mRNA levels above the level of controls is shown in Table 2. As expected the percentage of patients with mRNA values above controls increases with TNM-stage reaching a value of 41–100% in stage IV patients. Note, however, that 10–30% of stage I and II CC patients also show elevated levels for the biomarkers.

**Table 2:** Fraction of 166 CRC patients with biomarker mRNA expression levels in their highest lymph node above the level of the same biomarker mRNA in lymph nodes of control patients. Relation to TNM stage I-IV.

	Fraction of CRC patients (%) having lymph nodes with biomarker mRNA levels above the highest level of control lymph nodes			
Biomarker mRNA	Stage I	Stage II	Stage III	Stage IV
CEACAM5	57	59	41	100
KLK6	22	11	54	75
SLC35D3	18	9	25	79
MUC2	13	9	30	41
POSTN	25	13	32	69

### High levels of the biomarker mRNAs in lymph nodes is associated with poor prognosis

To determine whether high levels of any of the selected biomarker mRNAs was associated with poor prognosis we determined a cut-off using Cox's regression analysis as described [11]. Briefly, patients were divided into five groups according to the 20^th^, 40^th^, 60^th^ and 80^th^ percentile based on their biomarker mRNA levels. Each patient was represented by the lymph node with the highest level. Groups up to the 60^th^ percentile did not differ significantly from each other with regard to recurrence and were therefore combined. Patients in the 80^th^ percentile [= (biomarker(+) group] had an increased hazard risk ratio compared to those with values below the 80^th^ percentile [= biomarker(-) group]. An expression level cut-off at the 80^th^ percentile was valid for all five biomarker mRNAs. Table 3 shows the actual cut-off levels and hazard risk ratios for the two groups as well as the average survival times of the two groups as calculated by cumulative survival analysis according to Kaplan-Meier. Hazard risk ratios ranged from 2.48 to 4.67 for the five biomarkers and difference in survival time from 31 to 68 months, CEACAM5 and KLK6 showing the highest differences. All differences were highly significant.

**Table 3:** Average survival time and risk for recurrence of disease of CRC patients with biomarker(+) or biomarker(-) lymph nodes.

		Survival time after surgery			Risk for recurrence	
Biomarker	mRNA level* (copies/18S rRNA unit)	Average** (months)	Difference vs marker(-) (months)	*P*-value	Hazard ratio***	*P*-value
CEACAM5(-)	<4.2	112				
CEACAM5(+)	≥4.2	44	68	<0.0001	4.67	<0.0001
KLK6 (-)	<0.0831	110				
KLK6 (+)	≥0.0831	46	64	<0.0001	4.01	<0.0001
SLC35D3 (-)	<0.0059	103				
SLC35D3 (+)	≥0.0059	54	49	0.002	2.48	0.002
MUC2 (-)	<0.0045	108				
MUC2 (+)	≥0.0045	64	44	0.001	2.53	0.001
POSTN (-)	<11.05	107				
POSTN (+)	≥11.05	76	31	0.001	2.52	0.002

* The cut-off level is the 80^th^ percentile of the patient population.

** Mean survival time after surgery as calculated by cumulative survival analysis according to Kaplan-Meier.

*** Risk ratio as calculated according to univariate Cox's regression analysis.

### A formula for predicting prognosis of CRC patients based on all five biomarkers and lymph node biomarker mRNA ratios

**Fig 2:**
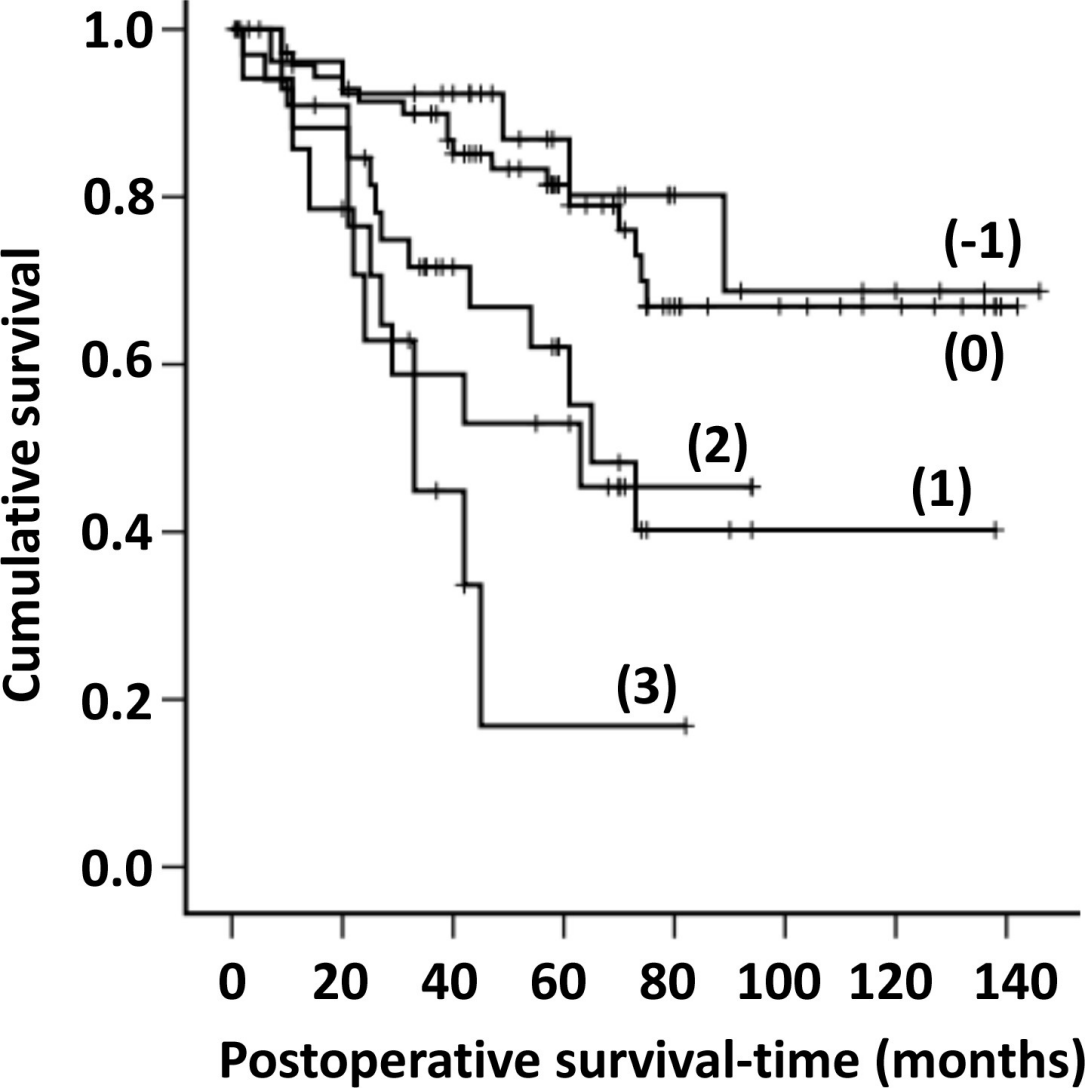
Cumulative survival curves according to Kaplan-Meier for CRC patients classified into groups (-1, 0, +1, +2, and +3) based on the mRNA values of the biomarkers CEACAM5, KLK6, SLC35D3, POSTN and MUC2 and calculated according to the formula (KLK6/CEACAM5+SLC35D3/CEACAM5+POSTN/18S rRNA-MUC2/CEACAM5). For KLK6 and SLC35D3 all mRNA levels above detection limit were considered as positive giving a value of (1) as opposed to no detectable mRNA giving a value of (0). For POSTN/18S rRNA and MUC2/CEACAM5 the 80th percentile of the rations was used to classify the value as positive or negative giving the former a value of (1) and the latter a value of (0).

Since measured biomarker mRNA values depend both on number of cells expressing the biomarker and biomarker level per cell it is reasonable to assume that a biomarker with relatively constant expression per cell could serve as the denominator in a ratio with a marker predicting aggressive qualities of the tumor cells. In the ratio calculations, CEACAM5 mRNA was used as the denominator because of its high and relatively constant expression levels per cell in CC tumor cells [12,22] and KLK6, SLC35D3 and MUC2 as the numerators because they predict aggressive (KLK6 and SLC35D3) and non-aggressive (MUC2) properties of cancer cells. For POSTN, which is expressed in fibroblasts and not in epithelial cells (Table 1), we used 18S rRNA as the denominator. The 80^th^ percentile of the ratios KLK6/CEACAM5, SLC35D3/CEACAM5, MUC2/CEACAM5 and POSTN/18S rRNA was used to assign nodes into one of two groups. Lymph nodes with a ratio above the 80^th^ percentile was given a value of (1) and below given a value of (0). When the following formula: [KLK6/CEACAM5 + SLC35D3/CEACAM5 + POSTN/18S rRNA -MUC2/CEACAM5] was applied each patient could be allocated to one of five groups (formula results: -1, 0, +1, +2, +3). Fig 2 shows the result of cumulative survival analysis according to Kaplan-Meier for each of these five groups and Table 4 summarizes the results. Categories (-1) and (0) show good 3- and 5-years survival, categories (+1) and (+2) relatively poor survival and category (+3) very poor survival. Table 4 also gives the result of hazard ratio calculations for the different categories using category (-1) as baseline in univariate Cox's regression analysis. The differences between the (+1 to +3) categories and the (-1) category are significant.

**Table 4:** Comparison between patients categorized according to biomarker expression, using formula A, i.e. [KLK6/CEACAM5 + SLC35D3/CEACAM5 + POSTN/18S rRNA -MUC2/CEACAM5], with regard to risk for recurrence of disease and cancer death and observed recurrence 3 and 5 years after curative surgery.

				Recurrence (%)**	
		Risk for recurrence		Time after surgery	
Category	n°	Hazard ratio*	*P*-value	3 years	5 years
-1	28	1.00		7	14
0	74	1.33	NS^#^	10	20
+1	33	3.15	0.028	28	37
+2	17	3.64	0.021	42	47
+3	14	6.98	0.001	56	84

** Percentage of patients in the indicated category who had died from CRC or lived with recurrent disease 3- and 5 years after surgery as determined by cumulative survival from Kaplan-Meier analysis.

° n = number of patients in the category.

* Calculated from the number of patients who had died from CRC or lived with recurrent disease 5 years after surgery using category -1 as baseline in univariate Cox's regression analysis.

^#^ NS = not statistically significant.

The results of multivariate survival analysis of prognostic factors of the CRC patients using Cox's proportional hazard regression model are shown in Tables 5 and 6. There was no significant difference with respect to the demographic parameters, i.e. sex and age, or site of lesion in either univariate or multivariate analysis (Table 5). A significant difference was however, seen for tumor grade (high versus low), TNM stage (I/II versus III/IV) and biomarker category (-1/0 versus 1/2/3) in univariate analysis, which persisted in multivariate analysis (Table 5), demonstrating that they were independent prognostic factors. Postoperative treatment, in contrast, was not an independent variable since significance disappeared completely when performing multivariate analysis. This is an expected finding because postoperative adjuvant or palliative chemotherapy is only offered to patients in the stage III/IV group. Table 6 shows the result of an extended multivariate analysis of biomarker categories in which we have excluded the variables that did not show a significant difference. Each biomarker category was compared with the category showing the best survival, i.e. the (-1) category. Significant differences were seen between the (-1) and (1) categories and between the (-1) and (3) categories.

**Table 5:** Multivariate survival analysis of demographic, clinical and prognostic factors for patients with CRC using Cox's proportional hazard regression model.

Variable	Univariate analysis			Multivariate analysis		
	n	Hazard ratio (95% CI)	*P*-value	n	Hazard ratio (95% CI)	*P*-value
Sex (male vs. female)	81/85	1.18 (0.69–2.02)	0.541	78/82	1.14 (0.64–2.03)	0.667
Age (<72 vs. ≥72 years)*	84/82	0.98 (0.57–1.67)	0.925	80/80	1.14 (0.55–2.38)	0.717
Site of lesion (colon vs. rectum)	153/13	0.76 (0.275–2.12)	0.605	148/12	1.16 (0.34–3.94)	0.807
Primary tumor grade (high vs. low)	23/137	0.37 (0.20–0.66)	0.001	23/137	0.50 (0.27–0.92)	0.026
TNM-stage (I/II vs. III/IV)	104/62	5.76 (3.23–10.30)	0.0001	101/59	4.25 (2.06–8.76)	0.0001
Postoperative adjuvant treatment (no vs. yes)	132/34	3.11 (1.79–5.39)	0.0001	129/31	0.98 (0.41–2.38)	0.969
Biomarker category (-1/0 vs. 1/2/3)	102/64	3.13 (1.81–5.42)	0.0001	97/63	1.85 (1.01–3.38)	0.045

n = number of individuals in the respective groups.

CI = confidence interval.

* Median age was 72 years.

**Table 6:** Multivariate survival analysis of the five biomarker categories of patients with CRC in relation to TNM-stage and primary tumor grade using Cox's proportional hazard regression model.

Variable	n	Hazard ratio (95% CI)	*P*-value
Primary tumor grade (high vs. low)	23/137	0.46 (0.25–0.84)	0.012
TNM-stage (I/II vs. III/IV)	101/59	4.09 (2.15–7.80)	0.0001
Biomarker category (-1 vs. 0)	27/70	1.93 (0.64–5.83)	0.244
Biomarker category (-1 vs. 1)	27/33	3.17 (1.038–9.67)	0.043
Biomarker category (-1 vs. 2)	27/17	2.50 (0.76–8.21)	0.132
Biomarker category (-1 vs. 3)	27/13	3.56 (1.02–12.43)	0.047

n = number of individuals in the respective category.

CI = confidence interval.

To determine whether all biomarker mRNAs were needed in order to achieve differentiation between the CRC patients into basically three categories with respect to risk for recurrence, one of the terms in the formula given above (A) was systematically excluded giving formula B, C, D, and E (S5 Table). Poorer discrimination between groups were seen in all four cases compared to determinations according to formula A (S5 and S6 Tables).

## Discussion

In this study we have selected a combination of five biomarkers which all have prognostic value. Two, SLC35D3 and POSTN, have not been used in lymph node analysis before. They describe different properties of CRC tumors and combined in a formula allocate CRC patients into 5 different risk groups. Importantly, none of the biomarker mRNAs are expressed in immune cells making them suitable for analysis of lymph nodes. KLK6 mRNA was chosen because it is ectopically expressed in CRC tumor in contrast to CEACAM5 and MUC2 that are expressed at similar levels in CRC tumors and normal colon. MUC2 was included because mucinous adenocarcinoma in CRC has been shown to have a better prognosis than adenocarcinoma in general [12,24]. CEACAM5 was selected because of its very high expression level and the observation that primary CRC tumors lacking CEACAM5 appear to be extremely rare [12,22,23]. SLC35D3 was chosen because it appears to represent another epithelial cell expression pattern than the other three biomarkers perhaps related to cellular immaturity.

The use of molecular methods for detection of tumor cells in lymph nodes has several advantages compared to the routine method for CRC, which is histopathology. Here an experienced pathologist examines H&E stained tissue sections for presence of tumor cells/tumor cell foci in ideally a few tissue sections per node. At least 12 lymph nodes should be analyzed in this way in order to determine whether the tumor has spread to the regional lymph nodes or not. Apart from the subjectivity of histopathology and that interpretation of the staining pattern require considerable experience it is labor intense. Moreover, there is the volume problem since only a small fraction of the volume of the lymph node is in fact investigated. Molecular methods like qRT-PCR on the other hand are objective, less labor intense, and allow analysis of the entire lymph node volume. Moreover, molecular methods are highly sensitive. While histopathology detects approximately 1 cancer cell/200 normal cells [25,26], qRT-PCR detects 1 cancer cell/ 10^6^ normal cells [22,27]. However, molecular methods are not without problems. Selection of biomarkers and establishment of a cut-off level for each biomarker must be carefully considered. Moreover, how to combine the recorded biomarker levels in a relevant but easily interpretable way to be helpful for the clinicians in deciding a treatment strategy is another important issue.

The high sensitivity of qRT-PCR constitutes a problem in that it is difficult to decide what is the best cut-off level for each marker. Is it the level where lymph nodes from CRC patients is higher than the highest level of lymph nodes from control patients such as patients with inflammatory bowel disease (Fig 1) or is it for example the level at the 80^th^ percentile of the patient population (Table 3)? This problem is akin to that faced in histopathology comparing prognosis for patients in which single tumor cells are found compared to lymph nodes with small or large (>0.2 mm) tumor foci. The latter having poorer prognosis than the other two [28–30]. In the study by Waldman et al., they demonstrated that as high a proportion as 87.5% of pN0 patients were positive for GUCY2C mRNA using the median level for this biomarker as cut-off [14]. Obviously, not all of pN0 succumb from their cancer. Our results for CEACAM5 mRNA using the highest level of control nodes as cut-off level are similar [12].

This problem was partly overcome by allowing traits of tumor cells that are related to aggressiveness, i.e. propensity to form distant metastases, play a more prominent role by calculating ratios for epithelial cancer cell derived markers. To this end we used CEACAM5 as the denominator for the three biomarkers KLK6, SLC35D3 and MUC2, that all reflect properties of the tumor cells. In this way complex data could be transformed into a simple formula that allowed allocation of patients to essentially three groups: patients with good, intermediate and poor 5-years disease-free survival. In the calculations the 80^th^ percentile was used as cut-off level for ratios. An optimal, precise cut-off might be settled upon analysis of a larger clinical material.

This study shows that, this transformation of data into a simple formula based on ratios for a few biomarkers has the potential of becoming an important adjunct to the classical method particularly as a basis for choice of postoperative treatment and follow-up regiments as well as being a helpful tool in future development of new therapies.

## Supporting information

S1 Table List of Taqman Gene Expression Assays used in qRT-PCR. (XLS)

S2 Table Genes with significantly higher mRNA expression levels in colon cancer tissue compared to mean mRNA signal of control colon tissue (P<0.05) as determined by genome-wide hydridization bead array. (XLS)

S3 Table Expression levels of CEACAM5, KLK6, POSTN, SLC35D3, PNCK, CLDN2, SULF1, FOXQ1, SERPINB5, CDH3, AZGP1, C16orf59 and C6orf223 mRNAs in a panel of CC and immune cell lines, colon epithelial cells, primary CC tumor tissue of 3 CC patients, normal colon tissue, and lymph nodes of 9 CC patients and 3 controls. (XLS)

S4 Table Correlation matrix between expression pattern in a panel of CC and immune cell lines, colon epithelial cells, primary CC tumor tissue of 3 CC patients, normal colon tissue, and lymph nodes of 9 CC patients and 3 controls of 13 different mRNA species. (DOCX)

S5 Table Percentage of CRC patients that have died from recurrent disease 3 and 5 years after surgery as determined by cumulative survival according to Kaplan-Meier. Comparison between patients classified into groups according to formula A, B, C, D and E.
(DOCX)

S6 Table Risk for recurrence of CRC after surgery as calculated according to univariate Cox regression analysis. Comparison between patients classified into groups according to formula B, C, D and E.
(DOCX)
