# Supplementary material for: Allocating colorectal cancer patients to different risk categories by using a five-biomarker mRNA combination in lymph node analysis
Source: PLoS One. 2020 Feb 12;15(2):e0229007. doi: 10.1371/journal.pone.0229007 (PMC7015415; doi:10.1371/journal.pone.0229007)
Supplement: S4 Table — (DOCX) [file pone.0229007.s004.docx]

**S4 Table**

Correlation matrix between expression pattern in a panel of CC and immune cell lines, colon epithelial cells, primary CC tumor tissue of 3 CC patients, normal colon tissue, and lymph nodes of 9 CC patients and 3 controls of 13 different mRNA species.

|  | CEACAM5 | KLK6 | SERPINB5 | FOXQ1 | CLDN2 | AZGP1 | C6orf223 | POSTN | SULF1 | PNCK | C16orf59 | SLC35D3 | CDH3 |
| --- | --- | --- | --- | --- | --- | --- | --- | --- | --- | --- | --- | --- | --- |
| CEACAM5 | 1.0 | 0.51  ** | 0.77  **** | 0.57  *** | 0.71  **** | 0.66  **** | 0.484  ** | 0.43  * | 0.25  ns | 0.18  ns | -0.04  ns | 0.39  * | 0.40  * |
| KLK6 |  | 1.0 | 0.69  **** | 0.72  **** | 0.65  **** | 0.65  **** | 0.521  ** | 0.06  ns | 0.11  ns | 0.19  ns | 0.34  ns | 0.55  ** | 0.67  **** |
| SERPINB5 |  |  | 1.0 | 0.68  **** | 0.71  **** | 0.76  **** | 0.49  ** | 0.35  ns | 0.24  ns | 0.18  ns | 0.17  ns | 0.44  * | 0.45  * |
| FOXQ1 |  |  |  | 1.0 | 0.84  **** | 0.67  **** | 0.21  ns | 0.32  ns | 0.32  ns | 0.12  ns | 0.08  ns | 0.37  * | 0.68  **** |
| CLDN2 |  |  |  |  | 1.0 | 0.66  **** | 0.33  ns | 0.19  ns | 0.19  ns | 0.36  * | 0.14  ns | 0.46  ** | 0.57  *** |
| AZGP1 |  |  |  |  |  | 1.0 | 0.50  ** | 0.29  ns | 0.21  ns | 0.01  ns | 0.11  ns | 0.43  * | 0.49  ** |
| C6orf223 |  |  |  |  |  |  | 1.0 | -0.07  ns | -0.09  ns | 0.20  ns | 0.50  ** | 0.15  ns | 0.29  ns |
| POSTN |  |  |  |  |  |  |  | 1.0 | 0.84  **** | -0.19  ns | -0.07  ns | 0.11  ns | 0.20  ns |
| SULF1 |  |  |  |  |  |  |  |  | 1.0 | -0.27  ns | -0.31  ns | -0.06  ns | 0.28  ns |
| PNCK |  |  |  |  |  |  |  |  |  | 1.0 | 0.46  ** | 0.29  ns | -0.21  ns |
| C16orf59 |  |  |  |  |  |  |  |  |  |  | 1.0 | 0.04  ns | 0.18  ns |
| SLC35D3 |  |  |  |  |  |  |  |  |  |  |  | 1.0 | 0.24  ns |
| CDH3 |  |  |  |  |  |  |  |  |  |  |  |  | 1.0 |

Amounts of the indicated mRNA species on which these calculations are based are shown in the **S3 Table**. Correlations are shown as r-values and significance as stars. *, *P*<0.05; **, *P*<0.01; ***, *P*<0.001and ****, *P*<0.0001 when comparing expression levels of indicated genes in the panel of cell types listed in **S3 Table**.
