# Supplementary material for: Allocating colorectal cancer patients to different risk categories by using a five-biomarker mRNA combination in lymph node analysis
Source: PLoS One. 2020 Feb 12;15(2):e0229007. doi: 10.1371/journal.pone.0229007 (PMC7015415; doi:10.1371/journal.pone.0229007)
Supplement: S5 Table — Comparison between patients classified into groups according to formula A, B, C, D and E. (DOCX) [file pone.0229007.s005.docx]

**S5 Table**

Percentage of CRC patients that have died from recurrent disease 3 and 5 years after surgery as determined by cumulative survival according to Kaplan-Meier. Comparison between patients classified into groups according to formula A, B, C, D and E.

|  | **Formula** | | | | | | | | | |
| --- | --- | --- | --- | --- | --- | --- | --- | --- | --- | --- |
|  | **A** | **B** | **C** | **D** | **E** | **A** | **B** | **C** | **D** | **E** |
| **Group** | **3 years** | | | | | **5 years** | | | | |
| -1 | 7 | 10 | 5 | - | 10 | 14 | 15 | 12 | - | 18 |
| 0 | 10 | 13 | 14 | 9 | 10 | 20 | 26 | 21 | 15 | 19 |
| +1 | 28 | 31 | 34 | 20 | 29 | 37 | 38 | 42 | 32 | 38 |
| +2 | 42 | 57 | 41 | 51 | 42 | 47 | 84 | 80 | 58 | 65 |
| +3 | 56 | - | - | 34 | - | 84 | - | - | 60 | - |

Formula A: [KLK6/CEACAM5+SLC35D3/CEACAM5+POSTN/18S rRNA -MUC2/CEACAM5] giving the groups-1, 0, +1, +2, +3

Formula B: [SLC35D3/CEACAM5+POSTN/18S rRNA-MUC2/CEACAM5] giving the groups -1, 0, +1, +2

Formula C: [KLK6/CEACAM5+POSTN/18S rRNA-MUC2/CEACAM5] giving the groups -1, 0, +1, +2

Formula D: [KLK6/CEACAM5+SLC35D3/CEACAM5+POSTN/18S rRNA] giving the group 0, +1, +2, +3

Formula E: [KLK6/CEACAM5+SLC35D3/CEACAM5-MUC2/CEACAM5] giving the groups -1, 0, +1, +2.
