# Supplementary material for: Allocating colorectal cancer patients to different risk categories by using a five-biomarker mRNA combination in lymph node analysis
Source: PLoS One. 2020 Feb 12;15(2):e0229007. doi: 10.1371/journal.pone.0229007 (PMC7015415; doi:10.1371/journal.pone.0229007)
Supplement: S6 Table — Comparison between patients classified into groups according to formula B, C, D and E. (DOCX) [file pone.0229007.s006.docx]

**S6 Table**

Risk for recurrence of CRC after surgery as calculated according to univariate Cox regression analysis. Comparison between patients classified into groups according to formula B, C, D and E.

|  | Formula B | | Formula C | | Formula D | | Formula E | |
| --- | --- | --- | --- | --- | --- | --- | --- | --- |
| Group | Risk of recurrence | | | | | | | |
|  | Hazards  ratio* | *P*-value | Hazards  ratio* | *P*-value | Hazards  ratio* | *P*-value | Hazards  ratio* | *P*-value |
| -1 | 1.00 |  | 1.00 |  |  |  | 1.00 |  |
| 0 | 0.49 | NS | 0.41 | NS# | 1.00 |  | 1.15 | NS |
| 1 | 2.34 | NS | 3.30 | 0.008 | 2.24 | 0.019 | 2.62 | 0.35 |
| 2 | 5.56 | 0.001 | 6.59 | <0.0001 | 4.91 | <0.0001 | 5.05 | 0.001 |
| 3 |  |  |  |  | 3.98 | 0.001 |  |  |

* Calculated from the number of patients who had died from CRC or lived with recurrent disease 5 years after surgery using category -1 or 0 as baseline in univariate Cox regression analysis.

^#^ NS = not statistically significant.

Formula B: [SLC35D3/CEACAM5+POSTN/18S rRNA-MUC2/CEACAM5] giving the groups -1, 0, +1, +2

Formula C: [KLK6/CEACAM5+POSTN/18S rRNA-MUC2/CEACAM5] giving the groups -1, 0, +1, +2

Formula D: [KLK6/CEACAM5+SLC35D3/CEACAM5+POSTN/18S rRNA] giving the groups 0, +1, +2, +3

Formula E: [KLK6/CEACAM5+SLC35D3/CEACAM5-MUC2/CEACAM5] giving the groups -1, 0, +1, +2.
